# Prevalence and clinical course of pelvic pain among transmasculine individuals on gender-affirming testosterone: a systematic review and meta-analysis

**DOI:** 10.1016/j.eclinm.2026.104233

**Published:** 2026-10-05

**Authors:** Susanna Weidlinger, Angela Niggli, Mick van Trotsenburg, Katharina Feil, Constanze Weidlinger, Angela Vidal, Marietta Gulz, Janna Pape, Jessica Laue, Brigitte Leeners, Michael von Wolff, Tanya Karrer, Karin Camastral-Urech

**Affiliations:** a Department of Reproductive Endocrinology, University Hospital of Zurich, Zurich 8091, Switzerland; b Sigmund Freud Private University, Vienna 1020, Austria; c Department of Gynaecological Endocrinology and Reproductive Medicine, Medical University of Innsbruck, Innsbruck 6020, Austria; d Women’s Clinic, Hospital Thun, Thun 3600, Switzerland; e Division of Gynaecological Endocrinology and Reproductive Medicine, University Women’s Hospital, Inselspital Bern, Bern 3010, Switzerland; f Medical Library, University Library Bern, University of Bern, Bern 3012, Switzerland; g Women’s Clinic, Cantonal Hospital Winterthur, Winterthur 8401, Switzerland

**Keywords:** Transmasculine individuals, Transgender men, Gender-affirming hormone therapy, Testosterone, Pelvic pain, Chronic pelvic pain

## Abstract

**Background:**

Pelvic pain is increasingly recognised among transmasculine individuals receiving gender-affirming testosterone therapy, yet no systematic synthesis of prevalence or clinical course exists.

**Methods:**

We conducted the first systematic review and meta-analysis of pelvic pain in transgender individuals. We searched MEDLINE, Embase, Scopus, and CENTRAL from January 2013 to June 2026. Studies reporting prevalence or clinical course of pelvic pain in transmasculine individuals with or without testosterone therapy were included. Methodological quality was assessed using Joanna Briggs Institute checklists. Pooled prevalence estimates were calculated using random-effects models. PROSPERO registration: CRD420261278643.

**Findings:**

Eleven studies encompassing 4176 transmasculine individuals were included, of whom 2891 received testosterone therapy. Among those receiving testosterone, the pooled prevalence of pelvic pain was 39% (95% CI 23–55%), significantly higher than in cisgender women (5–25%). Prevalence was higher in surgical cohorts (50%, 95% CI 12–88%) than in non-surgical populations (28%, 95% CI 13–46%). New-onset pelvic pain was reported in 31% (95% CI 5–65%) of previously asymptomatic individuals after testosterone initiation. Critically, among individuals with preexisting pain, 72% (95% CI 62–81%) reported persistence, while only 26% (95% CI 18–34%) reported improvement or remission after testosterone initiation.

**Interpretation:**

Pelvic pain is common among transmasculine individuals and frequently persists despite testosterone therapy. These findings challenge the widespread assumption that androgenic hormonal transition is protective against pelvic pain and highlight the urgent need for proactive assessment and gender-affirming management in this population. Prospective studies with standardised pain assessment and investigation of hormonal, tissue-level, and psychosocial mechanisms are needed to develop evidence-based interventions.

**Funding:**

No funding was received.

**Research in context**

**Evidence before this study**

We searched MEDLINE, Embase, Scopus, and CENTRAL for studies published from Jan 1, 2013, to Jun 29, 2026, using terms related to transgender identities and pelvic pain. Studies published in English or German were eligible. Despite growing clinical recognition of pelvic pain among transmasculine individuals receiving gender-affirming testosterone therapy, no systematic review or meta-analysis has quantitatively synthesised prevalence estimates or clinical course. Available evidence consisted predominantly of small, retrospective, or cross-sectional studies with heterogeneous outcome definitions and limited control for confounding. The prevalence of pelvic pain in this population remained poorly defined, and whether testosterone therapy is protective against or predisposes to pelvic pain was unclear. This represents a critical knowledge gap for an underserved population with unique hormonal, anatomical, and psychosocial considerations.

**Added value of this study**

This is the first systematic review and meta-analysis to quantitatively synthesise the prevalence and clinical course of pelvic pain in transmasculine individuals receiving testosterone therapy. By integrating data from 11 studies encompassing 4176 transmasculine individuals, of whom 2891 were receiving testosterone therapy, we provide the first pooled prevalence estimates for pelvic pain (39%, 95% CI 23–55%), quantify new-onset symptoms after testosterone initiation (31%, 95% CI 5–65%), and characterise trajectories of preexisting pain (72% persistence vs. 26% improvement/remission). We demonstrate that pelvic pain is substantially more common than in cisgender women (5–25%) and frequently persists despite testosterone therapy, challenging the widespread assumption that androgenic hormonal transition is protective. We also identify substantial heterogeneity across study populations and highlight critical methodological limitations, including reliance on self-reported outcomes, lack of validated pain assessment tools, and retrospective designs. This synthesis establishes the clinical burden of pelvic pain in this population and identifies key research priorities.

**Implications of all the available evidence**

Pelvic pain affects more than one-third of transmasculine individuals on testosterone and frequently persists despite therapy (72%), challenging the widespread assumption that androgenic hormonal transition is protective. Clinicians should proactively assess pelvic pain in a gender-affirming context, counsel patients about realistic expectations, and maintain ongoing gynaecologic monitoring despite amenorrhea, as residual ovarian and endometrial activity may contribute to symptoms. Barriers to care—including provider knowledge gaps, discrimination, and gender dysphoria associated with pelvic examinations—must be actively addressed. The multifactorial aetiology likely involves testosterone-induced vaginal atrophy, residual ovarian activity, pelvic floor dysfunction, and psychosocial stressors including minority stress. Future research should prioritise prospective cohort studies with standardised, validated pain assessment tools and investigation of underlying mechanisms to identify modifiable risk factors and therapeutic targets. This evidence synthesis provides the foundation for evidence-based clinical guidelines and targeted interventions in an underserved population.

## Introduction

Chronic pelvic pain is a prevalent and debilitating condition, estimated to affect 5–25%^1^, ^2^, ^3^ of cisgender women worldwide. It is defined as pain perceived to originate from pelvic structures, typically lasting more than 6 months.^4^ Its aetiology is heterogeneous and often multifactorial, with common contributors including endometriosis, irritable bowel syndrome, interstitial cystitis, pelvic floor dysfunction, central sensitization and psychosomatic factors including depression, anxiety, and trauma history.^2^^,^^4^

Chronic pain conditions, including pelvic pain, are substantially more prevalent in females than in males, with sex differences emerging after puberty, suggesting a modulatory role for gonadal hormones.^5^, ^6^, ^7^, ^8^, ^9^ Sex hormones modulate pain perception through multiple pathways, including direct effects on peripheral and central nociceptive processing, descending inhibitory pain pathways, and inflammatory and neuroimmune signalling.^7^^,^^10^, ^11^, ^12^ Estradiol exerts concentration-dependent and context-dependent effects on nociception, acting as antinociceptive at higher concentrations and pronociceptive at lower concentrations.^13^, ^14^, ^15^ Progesterone has been associated with antinociceptive properties, although the relationship is complex.^16^, ^17^, ^18^ Testosterone has been associated with antinociceptive effects in animal models,^19^^,^^20^ and observational data suggest an inverse association between endogenous testosterone levels and chronic pain prevalence in humans.^21^^,^^22^ However, a recent systematic review and meta-analysis found no consistent effect of androgens on experimental pain sensitivity,^23^ and intervention trials have yielded conflicting results,^24^^,^^25^ suggesting that the relationship between testosterone and pain perception in humans is more complex than animal models imply.

For transmasculine individuals—persons assigned female at birth who identify along the masculine spectrum—gender-affirming testosterone therapy raises testosterone concentrations into the physiologic cisgender male range while reducing endogenous estradiol concentrations to the cisgender male range.^26^ This hormonal transition has well-documented effects on the reproductive tract, including menstrual suppression,^27^^,^^28^ vaginal epithelial atrophy,^29^^,^^30^ and alterations in pelvic floor function,^31^ which may increase susceptibility to dyspareunia and pelvic pain.^32^ Importantly, testosterone does not reliably suppress ovarian activity or ovulation,^26^^,^^33^, ^34^, ^35^ and the endometrial response is variable, with both atrophic and proliferative patterns reported.^35^, ^36^, ^37^ Residual ovarian and endometrial activity may contribute to pelvic symptoms despite amenorrhea.

Pelvic pain has emerged as a clinically significant concern in this population, with retrospective data suggesting that more than one-third of transmasculine individuals on testosterone report pelvic pain, and that new-onset symptoms after testosterone initiation are common.^38^ A recent systematic review of gynaecologic function in transmasculine individuals identified pelvic pain as a frequently reported symptom with incompletely understood aetiologies.^32^ Additionally, transmasculine and gender-diverse individuals have been found to experience chronic pain and nociplastic symptoms at high rates, with psychosocial stressors identified as key contributors.^39^

Whether the shift from an oestrogenic to an androgenic hormonal milieu confers net protection against or predisposition to pelvic pain remains unclear. The antinociceptive properties of testosterone^23^^,^^24^ may be counterbalanced by the loss of oestrogen-mediated tissue trophism,^29^^,^^40^ residual ovarian and endometrial activity,^34^^,^^35^ and the complex interplay of neuroimmune and psychosocial factors unique to this population—including gender dysphoria, minority stress, and barriers to medical care.^41^, ^42^, ^43^

Despite growing clinical recognition and emerging data suggesting that pelvic pain affects a substantial proportion of transmasculine individuals, no systematic review or meta-analysis has quantified the prevalence or characterised the clinical course of pelvic pain in this population. This systematic review and meta-analysis aimed to address this gap by synthesising the available evidence on the prevalence, new-onset symptoms, and trajectories of preexisting pelvic pain among transmasculine individuals receiving gender-affirming testosterone therapy.

## Methods

### Protocol registration and reporting

This systematic review and meta-analysis was conducted according to the Preferred Reporting Items for Systematic Reviews and Meta-Analyses (PRISMA) guidelines^44^ and was part of a broader systematic review on chronic pain in transgender and gender-diverse people, prospectively registered with the International Prospective Register of Systematic Reviews (PROSPERO) (CRD420261278643).^45^ Given the breadth and clinical heterogeneity of the identified evidence, the present analysis was restricted to pelvic pain in transmasculine individuals; findings on other pain types and populations will be reported separately.

### Definitions

Transmasculine refers to individuals assigned female at birth who identify along the masculine spectrum, including transgender men, non-binary individuals with masculine gender identity or expression, and other gender-diverse individuals on the masculine spectrum. Transfeminine refers to individuals assigned male at birth who identify along the feminine spectrum, including transgender women, non-binary individuals with feminine gender identity or expression, and other gender-diverse individuals on the feminine spectrum.

### Eligibility criteria

Studies were eligible if they reported prevalence, incidence, or clinical course of pelvic pain in transmasculine or transfeminine individuals of any age receiving gender-affirming hormone therapy (GAHT) or no hormone therapy. GAHT was defined as testosterone, with or without gonadotropin-releasing hormone (GnRH) analogues, in transmasculine individuals, and as oestrogen, with or without GnRH analogues or antiandrogens, in transfeminine individuals.

Although the search strategy was designed to identify studies in both transmasculine and transfeminine populations, no studies reporting pelvic pain outcomes in transfeminine individuals were identified. Therefore, this review reports exclusively on transmasculine individuals.

For studies with mixed exposure populations, cohorts were categorised based on predominant hormone therapy if at least 85% of participants received the same treatment. This approach accepts that up to 15% of participants within a cohort may have received a different exposure, resulting in potential exposure misclassification for those individuals. Studies not meeting this 85% threshold were excluded unless stratified data by exposure were available.

Given the expected paucity of evidence on this topic, eligibility criteria were intentionally broad. All study designs reporting original data were eligible, including observational studies, case series, and qualitative studies. Case reports, conference abstracts, editorials, narrative reviews, and opinion pieces were excluded. Studies published in English or German were included.

Outcomes of interest were (1) prevalence of pelvic pain with and without GAHT, (2) proportion of new-onset pelvic pain following GAHT initiation, and (3) change in preexisting pelvic pain following GAHT initiation (improvement, persistence, or worsening).

### Search strategy

An information specialist developed the search strategy in collaboration with content experts using a structured and iterative approach. Seven key papers meeting inclusion criteria were identified by a content expert and used to develop and test the full search strategy. The strategy incorporated two core concepts: (1) transgender identities and (2) pain-related terms, excluding broader terms such as ache, spasm, or discomfort because they reduced the conceptual specificity of the search. Free-text terms (searched across titles, abstracts, and author keywords) were combined with controlled vocabulary (Medical Subject Headings [MeSH] terms). Animal-only studies and undesired publication types were excluded. Results were limited to publications from 2013 onward, corresponding to replacement of gender identity disorder with gender dysphoria in the Diagnostic and Statistical Manual of Mental Disorders (Fifth Edition) (DSM-5).^46^

The search strategy was reviewed by the research team, tested against the seven key papers and refined iteratively to optimise sensitivity and specificity. The final strategy was adapted for MEDLINE, Embase, Scopus, and the Cochrane Central Register of Controlled Trials (CENTRAL), accounting for database-specific indexing while preserving conceptual equivalence. Searches were initially conducted on Dec 9, 2025, and updated on June 29, 2026. Search results from all databases were exported and deduplicated using Deduklick software^47^ and imported into Covidence^48^ for screening. Additional duplicates not identified by Deduklick software^47^ were removed by Covidence.^48^ Full database-specific search strategies, search dates, database coverage, and numbers of records retrieved are provided in the Supplement.

### Study selection

The search identified 3548 records. After duplicate removal, 2252 unique records underwent screening. Title and abstract screening as well as full-text assessment were performed by 5 authors (N.A., W.C., V.A., G.M., C.U.K.), with each record independently reviewed by 2 reviewers using Covidence.^48^ Disagreements were resolved through consultation with a sixth reviewer (W.S.). A total of 212 articles proceeded to full-text assessment. Of these, 201 were excluded: 126 because they reported outcomes that did not meet the eligibility criteria, 46 because the study population did not meet the eligibility criteria, 21 because the intervention did not meet the eligibility criteria, six because of an ineligible publication type, and two because they reported the same study population as another study. Eleven studies were included in the review (Fig. 1). Studies were included if they (1) were clinical studies published in English or German, (2) reported prevalence data on pelvic pain in transgender individuals, and (3) documented the type of GAHT, including studies where no GAHT was administered (PRISMA flow diagram, Fig. 1).

**Fig. 1:**
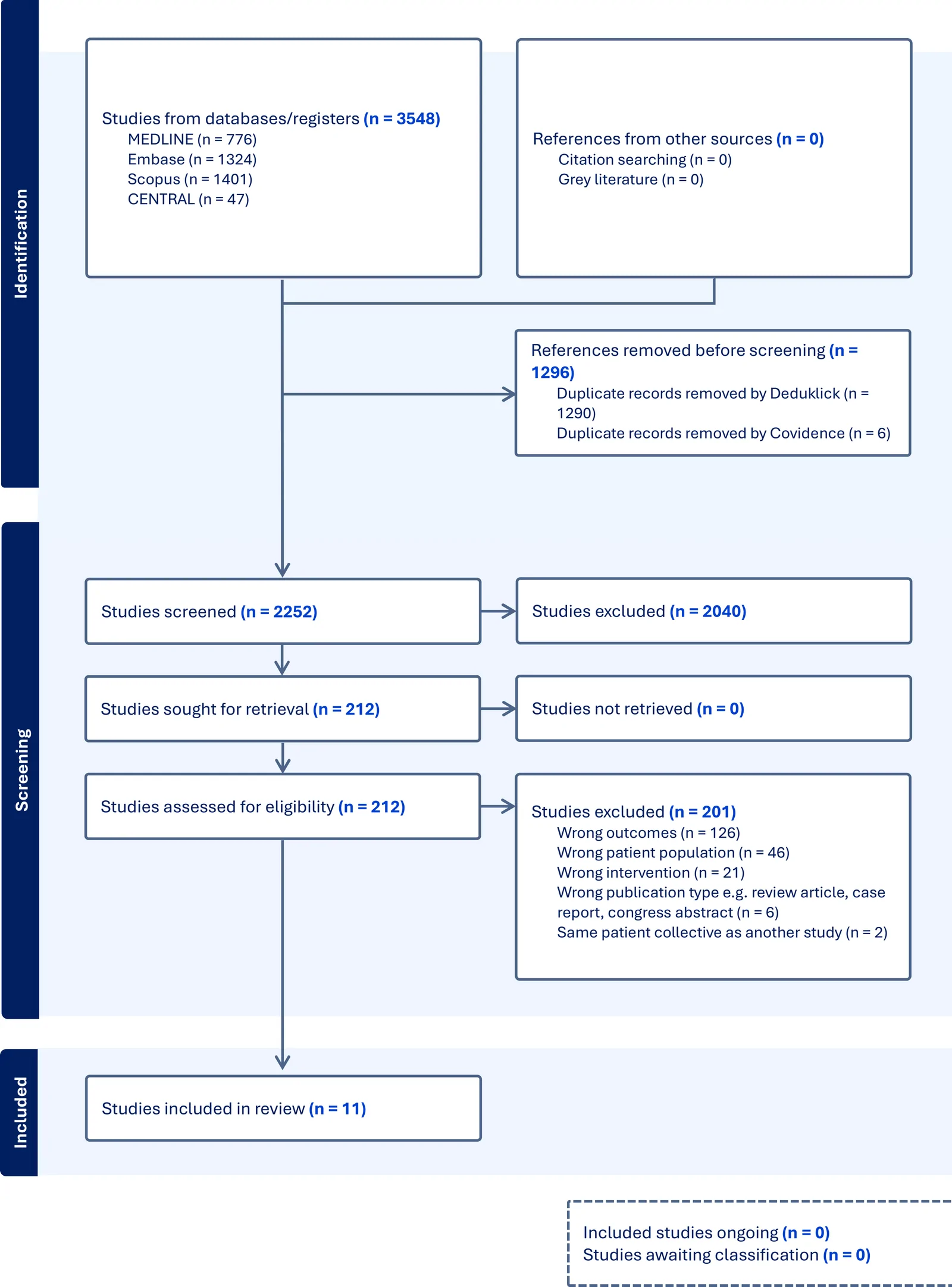
Preferred reporting items for systematic reviews and meta-analyses (PRISMA) flow diagram for new systematic reviews which included searches of databases and registers only.

### Data extraction

Two reviewers (W.S., C.U.K.) independently extracted data on study characteristics (design, country, recruitment period, sample size), participant characteristics (gender identity, age, scheduled gender-affirming surgery status), GAHT details (hormone type, treatment duration), and pelvic pain outcomes (prevalence, clinical course, pain type, assessment method). Discrepancies were resolved through discussion and consensus.

Operationalisation of pelvic pain: Given the anticipated heterogeneity in pain definitions across studies, we operationalised “pelvic pain” broadly to include any pain reported by participants or documented in medical records as being localised to the pelvis, lower abdomen, or pelvic organs, regardless of duration, cyclicity, or specific aetiology. We extracted detailed information on pain definitions, assessment methods, localisation, temporal characteristics (cyclic vs. non-cyclic, persistent vs. episodic), provocation/triggers, and pain subtypes (e.g., dysmenorrhea, dyspareunia, bladder pain, bowel pain) were reported. However, most studies did not provide standardised definitions or distinguish between pain subtypes. The majority of studies reported pelvic pain extracted from medical records or through self-report in online surveys, without validated pain assessment tools or differentiation between pain mechanisms (e.g., nociceptive, neuropathic, nociplastic). This heterogeneity in outcome definitions limits comparability across studies and the precision of pooled estimates.

### Data synthesis and statistical analysis

Prevalence estimates were calculated as the number of individuals reporting pelvic pain divided by the total number in each exposure group. Pooled estimates were computed using the Freeman-Tukey double arcsine transformation within a random-effects model, which stabilises the variance of proportions and yields approximately normally distributed observations. The conservative variance 1/(4n + 2) was applied as part of this transformation, which yields smaller within-study variance estimates and thereby proportionally increases the contribution of between-study heterogeneity to total variance. This approach is recommended for proportion meta-analyses, particularly when study numbers are small or proportions approach boundary values. Study weights under the random-effects model using the Freeman–Tukey double-arcsine transformation (W.(rFT)) are displayed in all forest plots. Results from untransformed proportion meta-analyses (random-effects model) are reported as sensitivity analyses in the Supplement. Heterogeneity was quantified using the Cochran Q statistic and I^2^ statistic. Prespecified subgroup analyses were conducted by testosterone use and scheduled gender-affirming surgery status to explore potential sources of heterogeneity. All analyses were performed using the metafor package (version 4.4.3) in R (version 4.4.3; R Foundation for Statistical Computing, Vienna, Austria).

### Quality assessment

Methodological quality was assessed using Joanna Briggs Institute (JBI) Critical Appraisal Checklists,^49^ appropriate to study design. Each item was scored as “yes” (1 point), “no” (−1 point), or “unclear” (0 points). Items deemed not applicable to the specific study design or research question were excluded from the total score calculation rather than assigned a numerical value.

For each study, a total quality score was calculated as the proportion of fulfilled applicable criteria and expressed as a percentage. Studies were categorised as low quality (0–59%), moderate quality (60–85%), or high quality (86–100%).

Quality assessments were conducted independently by two reviewers (S.W., C.W.), with discrepancies resolved through discussion and consensus.

### Role of the funding source

No funding was received for this work.

## Results

### Study selection and characteristics

The systematic search identified 11 studies from 5 countries (United States, 6; Australia, 3; Netherlands, 1; United Kingdom, 1; Spain, 1) that met inclusion criteria (Table 1). Studies were conducted between 2000 and 2024 and published between 2017 and 2026. Study designs included prospective cohort (n = 1),^51^ retrospective cohort (n = 5),^38^^,^^50^^,^^54^^,^^58^^,^^59^ cross-sectional (n = 3),^53^^,^^55^^,^^56^ retrospective case series (n = 1),^57^ and qualitative interview (n = 1)^52^ studies. The 11 studies enrolled a total of 4176 transmasculine individuals, with sample sizes ranging from 14 to 2579 participants (Tables 1 and 2).

**Table 1:** Overview of included studies, including country, study design, recruitment period, data sources, and definitions of pelvic pain.

Author, publication year	Country	Study design	Recruitment/data collection period	Data source/assessment method	Pelvic pain definition
Giacomozzi, 2026^50^	Netherlands & United Kingdom	Retrospective multicentre cohort study	2021–2024	Medical records	Pelvic pain not specifically defined
Tordoff, 2025^51^	United States of America	Prospective cohort study	2020–2022	Online survey, validated questionnaire	Chronic pelvic pain: persistent or recurrent pelvic pain in the pelvis, groin or genitals lasting three months or longer
Youngstrom, 2025^52^	United States of America	Single-centre, qualitative interview study	2021–2022	Semi-structured qualitative interviews	Chronic bladder pain
Moussaoui, 2024 (a)^53^	Australia	Cross-sectional study	2022–2023	Online survey, nonvalidated questionnaire	Pelvic pain: cyclical and acyclical abdominal pain in the lower half of the abdomen during the previous six months
Moussaoui, 2024 (b)^54^	Australia	Retrospective single-centre cohort study	2007–2020	Medical records	Pelvic pain, abdomino-pelvic pain or pain in the lower part of the abdomen
Borrás, 2024^55^	Spain	Single-centre cross-sectional study	2016–2021	Medical records	Chronic pelvic pain not specifically defined
Zwickl, 2023^56^	Australia	Cross-sectional study	2020–2020	Online survey, nonvalidated questionnaire	Pelvic pain not specifically defined
Grimstad, 2023^38^	United States of America	Retrospective single-centre cohort study	2002–2021	Medical records	Pelvic pain including but not limited to pain termed as cramps, dysmenorrhea, or discomfort, which patients identified as being in their pelvis
Cao, 2021^57^	United States of America	Retrospective single-centre case series study	2016–2020	Medical records	Pelvic pain not specifically defined as indication for surgery
Ferrando, 2021^58^	United States of America	Retrospective multicentre cohort study	2010–2019	Medical records	Constant and cyclic pelvic pain as indication for surgery
Obedin-Maliver, 2017^59^	United States of America	Retrospective single-centre cohort study	2000–2012	Medical records	Pelvic pain not specifically defined as indication for surgery

**Table 2:** Summary of study populations, exposure to gender-affirming hormone therapy, prevalence and clinical course of pelvic pain, and participant age characteristics.

Author, publication year	Study population	Gender-affirming hormone therapy	Participants with pelvic pain	Age (years)
Giacomozzi, 2026^50^	n = 325 transmasculine individuals scheduled for laparoscopic pelvic surgery	n = 310/325 (95.4%) on testosterone n = 10/325 (3.1%) not on testosterone n = 5/325 (1.5%) with missing data on testosterone use Duration of testosterone use: n = 278/299 (93.0%) ≥ 2 years	n = 17/325 (5.2%) with (chronic) PP	27.5 ± 9.1 (mean ± SD) 18–69 (range)
Tordoff, 2025^51^	n = 2579 transmasculine individuals	n = 1334/2579 (51.7%) testosterone current use n = 946/2579 (36.7%) testosterone never use n = 122/2579 (4.7%) testosterone former use n = 177/2579 (6.9%) with missing data on testosterone use Duration of testosterone use: median: 4 years, IQR: 2–7 years, range: 19 days to >30 years	Cross sectional data: n = 217/1334 (16.3%) of testosterone current users with chronic PP n = 180/946 (19.0%) of testosterone never users with chronic PP n = 28/122 (23.0%) of testosterone former users with chronic PP Longitudinal analyses among participants who were testosterone naive at baseline, and reported initiating testosterone at a consecutive survey: n = 5/9 (55.6%) with persistent chronic PP in the second year of testosterone use n = 7/66 (10.6%) with new onset chronic PP after testosterone initiation	27.6 (median) 23–34 (IQR) 18–80 (range)
Youngstrom, 2025^52^	n = 14 transmasculine individuals	n = 12/14 (85.7%) on testosterone	n = 5/14 (35.7%) with chronic bladder pain	30 ± 5.67 (mean ± SD)
Moussaoui, 2024 (a)^53^	n = 102 transmasculine individuals	n = 62/102 (60.8%) on testosterone n = 40/102 (39.2%) not on testosterone Duration of testosterone use: n = 49/62 (79%) ≥ 1 year	n = 36/40 (90%) with chronic PP not on testosterone n = 43/62 (69.4%) with chronic PP on testosterone n = 35/42 (83.3%) with chronic PP on testosterone + chronic PP already present before testosterone initiation n = 9/42 (21.4%) with chronic PP on testosterone + chronic PP improved after testosterone initiation n = 10/42 (23.8%) with chronic PP on testosterone + chronic PP worsened after testosterone initiation n = 7/42 (16.7%) with chronic PP on testosterone + chronic PP first occurred after testosterone initiation	18.1 ± 2.3 (mean ± SD)
Moussaoui, 2024 (b)^54^	n = 158 transmasculine adolescents on testosterone	n = 158/158 (100%) on testosterone Median follow-up after testosterone initiation: 26.4 months, range: 8.3–45.5 months in patients with pelvic pain 19.3 months, range: 6.1–57.4 months in those without pelvic pain	n = 37/158 (23.4%) with PP	–
Borrás, 2024^55^	n = 60 transmasculine individuals scheduled for gender-affirming surgery (hysterectomy + bilateral oophorectomy)	n = 60/60 (100%) on testosterone Duration of testosterone use: >24 months	n = 13/60 (21.7%) with chronic PP	26.7 (median) 23–34 (IQR)
Zwickl, 2023^56^	n = 486 transmasculine individuals	n = 486/486 (100%) on testosterone Duration of testosterone use: median: 32 months, range: 14.0–60.5	n = 35/486 (7.2%) with PP always or almost always	27 (median) 23–34 (IQR)
Grimstad, 2023^38^	n = 280 transmasculine individuals	n = 280/280 (100%) on testosterone Duration of testosterone use: median: 48 months, IQR: 27–251	n = 100/280 (35.7%) reported PP on testosterone n = 71/100 (71.0%) had no PP prior to testosterone n = 29/100 (29.0%) had PP prior to testosterone n = 42/280 (15.0%) had PP prior to testosterone n = 13/42 (31.0%) showed remission of PP after testosterone initiation n = 29/42 (69.0%) had persistent PP on under testosterone	–
Cao, 2021^57^	n = 72 transmasculine individuals scheduled for gender-affirming surgery (hysterectomy ± bilateral salpingectomy or salpingo-oophorectomy)	n = 66/72 (91.7%) prior or current use of testosterone n = 63/72 (87.5%) current use of testosterone Duration of testosterone use: median: 48 months, range: 3 months–15 years, IQR: 2.75–6 years	n = 65/72 (90.3%) with PP as indication for hysterectomy	30 (median) 19–51 (range)
Ferrando, 2021^58^	n = 67 transmasculine individuals scheduled for gender-affirming surgery (hysterectomy ± bilateral salpingo-oophorectomy)	n = 60/67 (89.6%) on testosterone n = 5/67 (7.5%) on other hormone therapy Duration of testosterone use: median: 36 months, range: 12–300	n = 34/67 (50.7%) with PP	29 ± 8 (mean ± SD)
Obedin-Maliver, 2017^59^	n = 33 transmasculine individuals scheduled for hysterectomy for benigne conditions	n = 30/33 (90.9%) on testosterone Duration of testosterone use: range: 2 months–14 years	n = 28/33 (84.8%) with PP as indication for hysterectomy	35.2 ± 9.9 (mean ± SD)

IQR, interquartile range; PP, pelvic pain; SD, standard deviation.

Included studies varied substantially in study design, population selection, testosterone exposure, and definition and ascertainment of pelvic pain (Tables 1 and 2). Study populations ranged from highly selected surgical cohorts to large community-based samples recruited via online surveys. Age distributions were generally centred in young adulthood, with mean or median ages ranging from approximately 18 to 35 years where reported.

Testosterone exposure varied considerably. Several studies included exclusively participants receiving testosterone, whereas others included mixed populations with current, former, or no testosterone use. Duration of testosterone therapy, when reported, typically ranged from several months to multiple years, with median durations of approximately 2–4 years in several cohorts.

Prior hysterectomy status varied across studies. Some cohorts consisted exclusively of individuals with an intact uterus scheduled for gender-affirming hysterectomy, while others included mixed populations of individuals with and without prior hysterectomy. Several studies did not report prior surgical history, precluding stratification by uterine status.

The proportion of participants reporting pelvic pain varied markedly, with estimates ranging from 5% to 90%. Higher proportions were observed in surgical cohorts, where pelvic pain was documented as an indication for hysterectomy.

Pain was most commonly self-reported using nonstandardised survey instruments or extracted from medical records. No study used a validated pain assessment tool, and only a minority explicitly defined chronic pelvic pain using established time-based criteria (e.g., duration ≥6 months).

Notably, pelvic pain was not the primary outcome in several included studies but was reported as a secondary outcome or clinical indication for surgical intervention. This variation in study objectives and outcome ascertainment likely contributed to the substantial heterogeneity observed across analyses.

### Quality assessment

A total of 11 studies were assessed using JBI critical appraisal tools^49^ appropriate to their respective study designs (cohort, analytical cross-sectional, case series, and qualitative research). Detailed item-level assessments are provided in the Supplement.

Overall methodological quality ranged from moderate to high. Three studies achieved high quality, while eight were of moderate quality; no study was classified as low quality.

Across cohort and cross-sectional studies, most fulfilled key criteria related to valid and reliable measurement of exposures and outcomes, as well as appropriate statistical analysis. However, a consistent limitation was the lack of explicit strategies to address confounding, even when potential confounders were identified. In addition, reporting and handling of follow-up were incomplete in several cohort studies.

The single included case series demonstrated high methodological rigor, with complete reporting across all JBI domains. The single qualitative study showed moderate quality, with strong methodological congruity but limited reflexivity and insufficient consideration of the researcher’s influence on the research process.

Overall risk of bias was low to moderate, though the limited handling of confounding across several studies should be considered when interpreting the findings (Table 3).

**Table 3:** Risk of bias summary table.

JBI, Joanna Briggs Institute.

### Prevalence of pelvic pain

In the overall analysis of transmasculine individuals receiving testosterone therapy (k = 11 studies; n = 2891 participants), the pooled prevalence of pelvic pain was 39% (95% CI 23–55%) using a random-effects model with Freeman-Tukey double arcsine transformation, with study-specific estimates ranging from 5% to 90% and thus with considerable heterogeneity across studies (I^2^ = 99.5%; P < 0.001) (Fig. 2A).

**Fig. 2:**
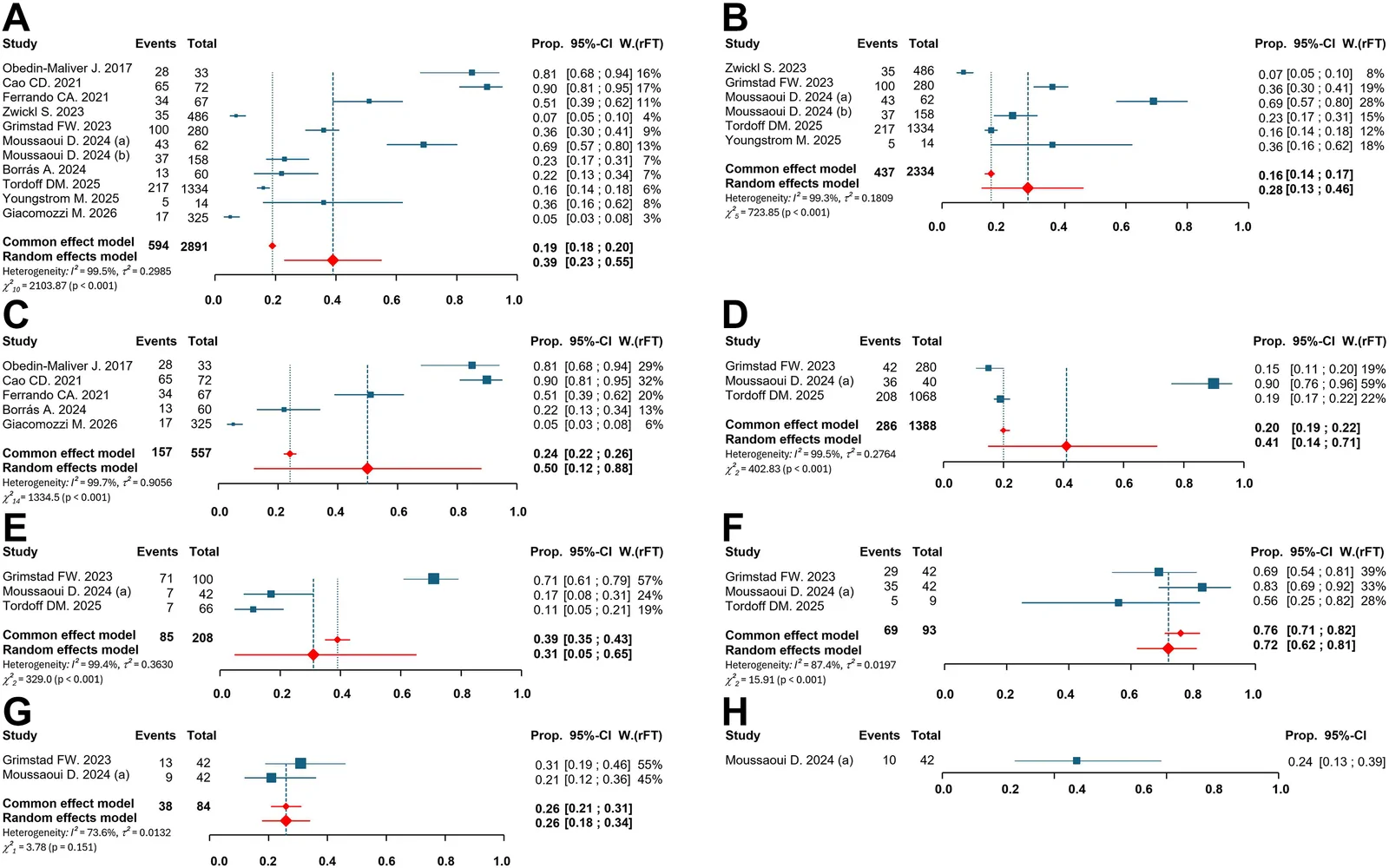
**(A)** Prevalence of pelvic pain among transmasculine individuals receiving testosterone therapy. **(B)** Prevalence of pelvic pain among transmasculine individuals receiving testosterone therapy, excluding those scheduled for gender-affirming surgery. **(C)** Prevalence of pelvic pain among transmasculine individuals receiving testosterone therapy and scheduled for gender-affirming surgery. **(D)** Prevalence of pelvic pain among transmasculine individuals not receiving testosterone therapy. **(E)** Proportion of new-onset pelvic pain after testosterone initiation among transmasculine individuals. **(F)** Prevalence of persistent pelvic pain after testosterone initiation among transmasculine individuals. **(G)** Prevalence of improved or remitted pelvic pain after testosterone initiation among transmasculine individuals. **(H)** Prevalence of worsened pelvic pain after testosterone initiation among transmasculine individuals. Squares represent study-specific prevalence estimates (proportions), with the size of each square reflecting the relative study weight; horizontal lines indicate the corresponding 95% confidence intervals (CIs). Diamonds represent the pooled estimates under the common-effect and random-effects models, with their widths indicating the corresponding 95% CIs. Between-study heterogeneity was quantified using the I^2^ statistic, the between-study variance (τ^2^), and Cochran’s Q test. Abbreviations: CI, confidence interval; I^2^, heterogeneity index; τ^2^, between-study variance; W.(rFT), study weight based on inverse variance after Freeman–Tukey transformation; Prop., proportion.

Given this heterogeneity, prespecified subgroup analyses were conducted according to testosterone use and scheduled gender-affirming surgery status (specifically hysterectomy with or without bilateral salpingo-oophorectomy). Among individuals receiving testosterone therapy not scheduled for gender-affirming surgery, the pooled prevalence of pelvic pain was 28% (95% CI 13–46%), with study-specific estimates ranging from 7% to 69% (I^2^ = 99.3%; P < 0.001) (Fig. 2B). Among individuals receiving testosterone therapy scheduled for gender-affirming surgery, the pooled prevalence was 50% (95% CI 12–88%), with estimates ranging from 5% to 90% (I^2^ = 99.7%; P < 0.001) (Fig. 2C).

Among individuals not receiving testosterone therapy, the pooled prevalence was 41% (95% CI 14–71%), with estimates ranging from 15% to 90% (I^2^ = 99.5%; P < 0.001) (Fig. 2D).

Stratification by surgery status and testosterone use did not substantially reduce heterogeneity, suggesting that additional factors—such as differences in pain definitions, assessment methods, or population characteristics—contribute to variability across studies. Pooled estimates should therefore be interpreted as descriptive summaries rather than precise population-level parameters.

### New-onset pelvic pain after testosterone initiation

Across 3 studies, the pooled prevalence of new-onset pelvic pain after testosterone initiation was 31% (95% CI 5–65%) using a random-effects model, with study-specific estimates ranging from 11% to 71% (I^2^ = 99.4%; P < 0.001) (Fig. 2E). Given differences in study design and methods for ascertaining symptom onset, these findings represent the proportion of individuals reporting new-onset symptoms rather than a precise incidence measure.

### Clinical course of preexisting pelvic pain

Among transmasculine individuals with preexisting pelvic pain, the pooled prevalence of persistent symptoms after testosterone initiation was 72% (95% CI 62–81%), with study-specific estimates ranging from 56% to 83% and thus substantial heterogeneity (I^2^ = 87.4%; P = 0.001) (Fig. 2F).

The pooled prevalence of improvement or remission was 26% (95% CI 18–34%), with substantial heterogeneity across studies (range 21–31%; I^2^ = 73.6%; P = 0.15) (Fig. 2G).

Data on worsening of chronic pelvic pain were limited to a single study, which reported worsening in 24% of participants (95% CI 13–39%) (Fig. 2H). No pooled estimate was calculated.

## Discussion

This systematic review and meta-analysis constitutes the first quantitative synthesis of the prevalence and clinical course of pelvic pain among transmasculine individuals receiving gender-affirming testosterone therapy. Across 11 studies encompassing 2891 participants, the pooled prevalence of pelvic pain among those receiving testosterone was 39%, with considerable heterogeneity across studies. Among individuals with preexisting pelvic pain, persistence of symptoms after testosterone initiation was more common than resolution (72% vs. 26%), and new-onset pelvic pain was reported in approximately 31% of previously asymptomatic individuals. These findings challenge the widespread assumption that testosterone uniformly confers protection against pelvic pain and underscore the clinical complexity of pain outcomes in this population.

The pooled prevalence of 39% among transmasculine individuals on testosterone exceeds the estimated 5–25% prevalence of chronic pelvic pain in cisgender women worldwide,^1^, ^2^, ^3^ although direct comparison is limited by differences in study populations, pain definitions, and ascertainment methods. Prevalence varied substantially by study population. The two largest community-based studies, which recruited participants through online transgender health cohorts, reported prevalence estimates of 7% and 16%,^51^^,^^56^ suggesting a lower but still clinically relevant burden in the broader transmasculine population, although these estimates remain subject to self-selection and recall bias. In contrast, prevalence was substantially higher in surgical cohorts (pooled prevalence 50%, 95% CI 12–88%), in which pelvic pain may itself have been an indication for gender-affirming hysterectomy.^57^, ^58^, ^59^ This finding is particularly relevant when considering the relationship between study quality and effect size: the three studies rated as high quality using JBI criteria reported prevalence estimates of 90%,^57^ 81%,^59^ and 69%,^53^ with the two highest estimates derived from surgical cohorts in which pelvic pain was documented as a surgical indication. This pattern permits two non-mutually exclusive interpretations: (1) methodologically rigorous studies with comprehensive symptom ascertainment may more accurately capture the true burden of pelvic pain, or (2) the high prevalence in surgical cohorts may reflect confounding by indication, with pelvic pain contributing both to the decision to undergo hysterectomy and to its ascertainment as an outcome. The substantially lower pooled prevalence in non-surgical populations (28%, 95% CI 13–46%) lends support to the latter interpretation.

Pelvic pain prevalence was similar among transmasculine individuals receiving and not receiving testosterone (39% vs. 41%, respectively), with substantially overlapping confidence intervals. These findings provide no evidence of a clear association between testosterone use and pelvic pain prevalence. However, this comparison should not be interpreted causally, as individuals receiving and not receiving testosterone may differ systematically with respect to dysphoria severity, menstrual status, access to care, or underlying gynaecologic conditions. The absence of randomised data and the cross-sectional nature of most included studies preclude any causal conclusions regarding the effect of testosterone on pelvic pain prevalence.

A recent systematic review reported a pooled endometriosis prevalence of 8.9% among transmasculine individuals across diverse study populations, with 53.9% of those affected reporting nonmenstrual pelvic pain.^60^ Given that the pooled prevalence of pelvic pain in the present analysis (39%) substantially exceeds the estimated prevalence of endometriosis, these findings suggest that pelvic pain in this population extends well beyond endometriosis as a single aetiology and is likely multifactorial in origin. Notably, the ETRIS multicentre cohort study reported a lower endometriosis prevalence of 3.1% among transmasculine individuals undergoing pelvic surgery, in a cohort characterised by prolonged testosterone exposure compared with prior studies, potentially attributable to sustained hormonal suppression and early initiation of gender-affirming care.^50^

Among the most clinically important findings of this review is the consistency of data on the clinical course of preexisting pelvic pain. The pooled prevalence of persistent symptoms after testosterone initiation was 72%, with substantial heterogeneity. In contrast, improvement or remission was reported in 26% of individuals, with substantial heterogeneity. These findings argue against a simplified narrative that testosterone typically resolves preexisting pelvic pain. Rather, the data suggest that while a subset of individuals experiences meaningful improvement, the majority continue to report symptoms despite testosterone therapy.

These findings are consistent with the observation by Grimstad et al. that individuals with pelvic pain on testosterone were significantly more likely to have had preexisting pain, dysmenorrhea, endometriosis, or ovarian cysts prior to testosterone initiation.^38^ This suggests that preexisting gynaecologic pathology may predispose to persistent pain despite hormonal transition.

The finding that 31% of previously asymptomatic individuals reported new-onset pelvic pain after testosterone initiation is also noteworthy, though the wide confidence interval and extreme heterogeneity (I^2^ = 99.4%) limit the precision of this estimate. The three studies contributing to this estimate differed substantially in design and methodology: Grimstad et al. used retrospective chart review in a clinical cohort (n = 100) and reported 71% new-onset pain^38^; Moussaoui et al. used cross-sectional self-report in adolescents (n = 42) and reported 17%^53^; and Tordoff et al. conducted a prospective cohort study with online surveys (n = 66) and reported 11%.^51^ This heterogeneity reflects differences in study design, population characteristics (clinical vs. community-based recruitment, adults vs. adolescents), and symptom ascertainment methods (chart review vs. self-report). The pooled estimate should therefore be interpreted with caution, as it may obscure clinically meaningful subgroup differences. The substantially higher estimate in the clinical cohort (71%) may reflect more systematic symptom documentation in medical records, higher baseline risk in treatment-seeking populations, or both.

The pathophysiology of pelvic pain in transmasculine individuals on testosterone is likely multifactorial and incompletely understood. Several mechanisms may contribute, either individually or in combination.

First, testosterone-induced vaginal atrophy,^29^^,^^30^ analogous to the genitourinary syndrome of menopause in cisgender women, may increase susceptibility to dyspareunia, bacterial vaginosis, and cystitis.^27^^,^^29^^,^^32^ Tordoff et al. reported that 64.6% of transmasculine individuals experienced vulvovaginal pain during sexual activity, with current testosterone use independently associated with increased vaginal pain.^40^

Second, residual ovarian and endometrial activity may contribute to ongoing pelvic symptoms. Despite amenorrhea and testosterone levels within the cisgender male range, histological signs of recent ovulatory activity have been documented in approximately 30–40% of transmasculine individuals at oophorectomy,^34^^,^^35^ and this finding was independent of testosterone serum levels, duration, or preparation type.^34^^,^^35^ Endometrial activity—whether proliferative or secretory—persists in a substantial proportion.^31^^,^^35^, ^36^, ^37^ Notably, a single large cohort study found that endometrial proliferation was significantly more frequent with transdermal gel and shorter-acting intramuscular testosterone esters (enanthate, cypionate; typically injected every 1–4 weeks) compared with the long-acting intramuscular testosterone undecanoate (injected every 10–14 weeks; OR 3.71 and 3.78, respectively).^35^ This finding awaits confirmation but is biologically plausible: shorter-acting esters produce supraphysiological peak levels followed by a decline into the low-normal range before the next injection, and transdermal gel may be subject to lower treatment adherence given its daily dosing schedule compared with the 10–14-week interval of testosterone undecanoate. Both scenarios may result in less effective suppression of ovarian function, with intermittent recovery of follicular activity and consequently increased local aromatisation of testosterone to estradiol, thereby sustaining endometrial stimulation.^35^ However, this residual endometrial and ovarian activity may sustain pelvic symptoms including pain. Of particular clinical relevance, endometriosis may persist or develop despite testosterone therapy. Ferrando et al.^58^ reported that 26.9% of transmasculine individuals undergoing gender-affirming hysterectomy had intraoperatively confirmed endometriosis—a substantially higher estimate than the pooled prevalence of 8.9% reported across mixed transmasculine populations,^60^ likely reflecting both selection bias in surgical cohorts and increased detection through direct surgical visualisation—with a higher prevalence among those reporting pelvic pain (32.3% vs. 21.9%), suggesting that endometriosis remains a relevant contributor to pelvic pain even in the setting of prolonged testosterone exposure.^58^

Third, pelvic floor dysfunction appears to be highly prevalent in this population. Da Silva et al. reported pelvic floor dysfunction in 94.1% of transmasculine individuals on testosterone.^31^ A biological basis is plausible: the levator ani—the principal muscle of the pelvic floor—is highly androgen-responsive, as evidenced by a significant volume loss under androgen deprivation in cisgender men^61^^,^^62^ and significantly increased pelvic floor electromyographic activity in women with androgen excess due to polycystic ovary syndrome,^63^ although this finding has not been consistently replicated.^64^ Whether masculinising testosterone doses induce pelvic floor hypertonicity in transmasculine individuals—potentially contributing to pelvic floor dysfunction and pain—has not been directly studied but warrants investigation. The relative contribution of testosterone-induced tissue changes vs. dysphoria-related behavioural patterns (such as habitual pelvic floor guarding) remains to be elucidated.^65^

Fourth, psychosocial factors unique to this population—including gender dysphoria, minority stress, and barriers to medical care—may amplify pain perception and reduce help-seeking behaviour.^41^^,^^66^ Merchant et al. demonstrated that stress, rather than gender identity or gender-affirming hormone therapy type, was the factor most strongly associated with chronic pain in gender minority individuals.^39^ This finding highlights the importance of a biopsychosocial framework in understanding pelvic pain in this population.

This review represents the first systematic synthesis of evidence on pelvic pain in transmasculine individuals receiving testosterone therapy. The review was conducted in accordance with PRISMA guidelines, with prespecified subgroup analyses to explore sources of heterogeneity. The inclusion of diverse study designs—from large community-based surveys to surgical cohorts—provides a broad perspective on pain prevalence across clinical contexts.

However, several limitations warrant consideration. First, the considerable heterogeneity observed across nearly all analyses (I^2^ > 90% for most pooled estimates) limits the interpretability of summary estimates. This heterogeneity reflects fundamental differences in study populations (surgical cohorts vs. community samples), pain definitions (clinical indication for surgery vs. self-reported chronic pain), assessment methods (medical records vs. online surveys), and testosterone exposure (duration, preparation, serum levels). Pooled prevalence estimates should therefore be interpreted as descriptive summaries of the available literature rather than precise population-level parameters.

Second, no included study used a validated, standardised pelvic pain assessment tool, and only a minority explicitly defined chronic pelvic pain using time-based criteria. Different pain subtypes—including cyclic vs. persistent pain, dyspareunia, and pain originating from different pelvic structures—are unlikely to respond uniformly to testosterone therapy. For example, endometriosis-associated pain may improve due to suppression of ovarian activity, whereas atrophy-related dyspareunia or pelvic floor dysfunction may be exacerbated by testosterone. The inability to distinguish between pain mechanisms limits identification of which patient subgroups are most likely to experience persistence, improvement, or worsening of symptoms, and precludes mechanistic conclusions. Subgroup analyses by pain subtype were not feasible. Additionally, most studies did not control for important confounders such as prior therapeutic interventions, surgical history, or concurrent gynaecologic conditions. This lack of standardisation and inadequate confounder control limit comparability across studies and may have contributed to the wide range of reported prevalence estimates (5–90%) and the extreme heterogeneity (I^2^ > 90% for most analyses).

Third, the cross-sectional design of most included studies precludes causal inference regarding the relationship between testosterone and pelvic pain, the impact of gender-affirming surgery, or the independent contribution of gender minority status and associated psychosocial stressors. The absence of cisgender comparison groups and pre-post surgical data limits the ability to isolate the effect of testosterone from other contributing factors. The single prospective cohort study^51^ included in this review provides the strongest evidence for temporal associations but is limited by its reliance on self-reported outcomes and potential for recall bias.

Fourth, confounding by indication is a concern in surgical cohorts, where pelvic pain may be both a reason for seeking hysterectomy and an outcome of interest. Additionally, reporting bias may inflate prevalence estimates in surgical cohorts. Although gender dysphoria alone is recognised as a medically necessary indication for gender-affirming hysterectomy by major professional societies including the American College of Obstetricians and Gynecologists (ACOG) and the World Professional Association for Transgender Health (WPATH),^26^^,^^29^ insurance coverage policies vary substantially across countries and payers. Many insurance companies require prior authorisation, mental health assessment letters, or additional documentation before approving gender-affirming surgery,^26^^,^^29^ and coverage criteria often exceed WPATH Standards of Care recommendations.^67^^,^^68^ In settings where coverage is uncertain or restrictive, clinicians and patients may be incentivised to document additional medical indications such as pelvic pain to facilitate insurance approval, potentially inflating the documented prevalence of pelvic pain in surgical cohorts beyond the true symptom burden. This phenomenon has been observed in other clinical contexts where insurance coverage depends on specific diagnostic criteria. The substantially higher prevalence of pelvic pain documented as a surgical indication in some cohorts (e.g., 90% in Cao et al.^57^) compared to community-based samples (7–16% in Tordoff et al.^51^ and Zwickl et al.^56^) may reflect, in part, this reporting bias rather than true differences in symptom prevalence. This likely inflates prevalence estimates in surgical subgroups.

Fifth, incomplete reporting of prior hysterectomy status across studies precluded stratification by uterine status, which may have influenced prevalence estimates. Critically, no studies reported pelvic pain outcomes before and after gender-affirming hysterectomy, precluding assessment of the impact of uterine and ovarian removal on pain trajectories.

Sixth, classifying studies by predominant hormone therapy when at least 85% of participants received the same treatment may have introduced exposure misclassification of up to 15%.

Seventh, the included studies were predominantly from the United States and Australia, limiting generalisability to other geographic and cultural contexts. The absence of studies from low- and middle-income countries is a notable gap.

Eighth, publication bias cannot be excluded, as studies reporting higher prevalence of pelvic pain may be more likely to be published. The small number of studies precluded formal assessment of publication bias.^69^

Ninth, the absence of studies on transfeminine individuals represents a significant gap in the literature. Although oestrogen-based hormone therapy and gender-affirming surgical procedures in transfeminine individuals may also influence pelvic pain through hormonal, anatomical, and psychosocial pathways, no studies meeting inclusion criteria were identified. This may reflect true differences in symptom burden, differential symptom reporting or recognition, underreporting due to stigma or lack of clinical recognition, or gaps in research prioritisation.

Based on the available evidence, the following recommendations can be made:

Pretreatment counselling: Clinicians should counsel transmasculine individuals that pelvic pain is common both before and during testosterone therapy, affecting approximately 40% of individuals, and that testosterone does not reliably resolve preexisting symptoms.^32^^,^^38^

Baseline assessment: Preexisting gynaecologic conditions should be evaluated and addressed prior to testosterone initiation when feasible, as these conditions are associated with increased risk of persistent or new-onset pelvic pain.^38^

Ongoing monitoring: Gynaecologic follow-up should continue despite amenorrhea, given the evidence of residual ovarian and endometrial activity that may contribute to pelvic symptoms.^32^

Low threshold for investigation: Clinicians should maintain a low threshold for investigating new or worsening pelvic pain, including consideration of endometriosis, pelvic floor dysfunction, and testosterone-induced vaginal atrophy.^32^^,^^40^

Affirming care environment: Clinicians should actively address barriers to medical care—including provider knowledge gaps, discrimination, and dysphoria associated with pelvic examinations—through inclusive language, trauma-informed approaches, and alternatives such as self-collected specimens.^29^^,^^41^ Individualised, gender-affirming care that integrates biopsychosocial factors, including minority stress and dysphoria, is essential for optimising outcomes in this population.^32^^,^^41^

Research priorities: Future studies should employ validated, multidimensional pain assessment tools that distinguish between pain subtypes and mechanisms. The recently validated Transmasculine and Gender Diverse Pelvic Symptom Index (Trans-PSI) represents an important advance, providing the first gender-affirming instrument specifically designed for this population.^70^ Differentiation between cyclic vs. non-cyclic pain, dyspareunia vs. non-sexual pelvic pain, and visceral vs. musculoskeletal pain is essential to identify which pain phenotypes are testosterone-responsive vs. those that may be exacerbated by testosterone, and to develop targeted interventions. Prospective cohort studies with standardised pain assessment at baseline (pre-testosterone), during testosterone therapy, and post-hysterectomy/oophorectomy are needed to characterise pain trajectories and identify modifiable risk factors for this population.

This systematic review and meta-analysis provides the first quantitative synthesis of pelvic pain in transmasculine individuals receiving testosterone therapy. Pelvic pain is common in this population, affecting 39% of individuals on testosterone, with higher rates in surgical cohorts (50%). Critically, testosterone does not reliably resolve preexisting pelvic pain: among individuals with preexisting symptoms, three-quarters (72%) report persistence after testosterone initiation, while only one-quarter (26%) experience meaningful improvement or remission. New-onset pelvic pain after testosterone initiation affects approximately one-third (31%) of previously asymptomatic individuals, and worsening of preexisting symptoms occurs in a subset.

These findings challenge the widespread assumption that androgenic hormonal transition confers uniform protection against pelvic pain. The pathophysiology is likely multifactorial, involving testosterone-induced vaginal atrophy, residual ovarian and endometrial activity despite amenorrhea, pelvic floor dysfunction, and psychosocial factors including minority stress and barriers to affirming medical care. Importantly, the high rate of pain persistence despite testosterone therapy underscores that psychosocial factors may perpetuate symptoms independently of hormonal status, consistent with the biopsychosocial model of chronic pelvic pain. Clinicians should counsel patients about realistic expectations, evaluate preexisting gynaecologic pathology prior to testosterone initiation when feasible, maintain ongoing monitoring despite amenorrhea, and address barriers to care through creation of gender-affirming clinical environments. Standardised assessment tools, prospective cohort studies, and mechanistic research are essential to improve understanding and management of this prevalent and clinically significant condition in an underserved population.

## Contributors

All authors had full access to all of the data in the study and take responsibility for the integrity of the data and the accuracy of the data analysis. W.S. and C.U.K. accessed and verified the underlying data. Conceptualisation: W.S., v.T.M., F.K., L.J. Methodology: W.S., v.T.M., F.K., L.J., K.T., P.J. Investigation (systematic literature search, screening, and data extraction): W.S., N.A., W.C., V.A., G.M., P.J., K.T., C.U.K. Formal analysis: P.J., W.S. Data curation: W.S., N.A., C.U.K. Validation (quality assessment and data verification): W.S., W.C. Writing—original draft: W.S. Writing—review and editing: All authors. Visualisation: W.S., P.J. Project administration: W.S., N.A., C.U.K. Supervision: v.T.M., F.K., L.B., v.W.M. All authors read and approved the final version of the manuscript.

## Data sharing statement

All data extracted from included studies are available in the manuscript and Supplementary materials. The full dataset is available from the corresponding author upon reasonable request.

## Declaration of interests

FK has received payment for lectures from FOMF and Trans-X, payment for a CME article from Springer, and support for attending the ESHRE meeting from Astropharma. vTM has received royalties from Cambridge University Press for the book TransGynecology and serves as unpaid Chairman of the Board of “Menschen für Menschen”, a non-governmental organization focused on sustainable development aid in rural Ethiopia. All other authors declare no financial or non-financial interests relevant to the submitted work.
