# Supplementary material for: Prevalence and clinical course of pelvic pain among transmasculine individuals on gender-affirming testosterone: a systematic review and meta-analysis
Source: eClinicalMedicine. 2026 Oct 5;100:104233. doi: 10.1016/j.eclinm.2026.104233 (PMC13671330; doi:10.1016/j.eclinm.2026.104233)
Supplement: Supplementary file 1 [file mmc1.pdf]

SUPPLEMENTARY MATERIAL

Prevalence and clinical course of pelvic pain among transmasculine individuals on gender-affirming testosterone: A systematic review and meta-analysis

Consolidated supplementary file

Table of contents

Search documentation	pages 3 – 8
Sensitivity analysis	pages 9 – 10
Quality assessment	pages 11 – 14

Search Strategies

Pain in Transgender People

- OVERVIEW -

Search date	Database	Platform Provider	Database Coverage	Results before deduplication	Results after deduplication
June 29, 2026 (Dec 9, 2025)	MEDLINE(R) ALL	Ovid (Wolters Kluwer)	1946 – Jun 26, 2026 (1946 – Dec 8, 2025)	776 (673)	760 (672)
June 29, 2026 (Dec 9, 2025)	Embase	Ovid (Wolters Kluwer)	1974 – Jun 25, 2026 (1974 – Dec 05, 2025)	1324 (1191)	790 (711)
June 29, 2026 (Dec 9, 2025)	Scopus	Elsevier	2004 – present (2004 – Dec 9, 2025)	1401 (1806)	1044 (977*)
June 29, 2026 (Dec 9, 2025)	Cochrane Register of Controlled Trials (CENTRAL)	Wiley	1996 – present (1996 – Dec 9, 2025)	47 (43)	22 (19)
**Total**				3548 (3713)	**2616** (2021**)
Original search (Dec 9, 2025)					
* Records published before 2013, removed manually (EndNote): -358					
** Records after year correction: 2021					
Duplicates identified at import by Covidence: -5					
Records screened in original review: 2016					
Update search (June 29, 2026)					
Records already identified in the original search (Dec 9, 2025): 2379					
Newly identified records (June 29, 2026): 237					
Duplicates identified at import by Covidence: 1					
New records screened: 236					
**Total records screened (original + update): 2252**					

* In the original search (9 December 2025), the publication year limit was not correctly applied in Scopus, so the initial set included records published before 2013. After deduplication, 358 records published before 2013 were identified and removed manually in EndNote. This affected the Scopus results only; the year limit was applied correctly in all other databases and in the updated search (26 June 2026).

Search update deduplication report

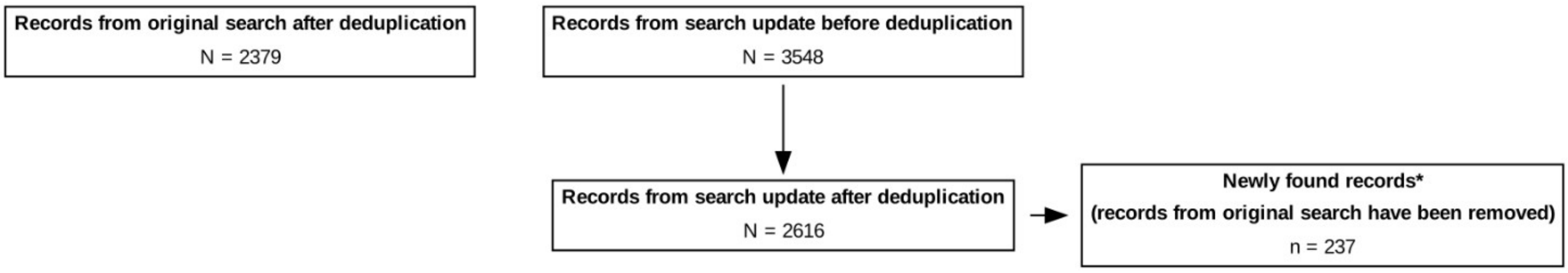

* The newly found records to screen are NOT the exact difference between the deduplicated records of the update search and the deduplicated original search. This discrepancy is the consequence of normal database activities like updates to individual records or changes to controlled vocabulary.See more detailed information about how to correctly represent search update numbers in the PRISMA flow chart on page 288 of the following publication:
Bramer W, Bain P. Updating search strategies for systematic reviews using EndNote. J Med Libr Assoc. 2017 Jul;105(3):285-289. doi: 10.5195/jmla.2017.183

Update flow chart from Deduklick update tool: https://www.risklick.ch/deduklick.

- SEARCHES -

MEDLINE/Ovid

Search date: June 29, 2026

Ovid MEDLINE(R) ALL <1946 to June 26, 2026>		
1	(transident* or trans ident* or transgender* or trans gender* or gender divers* or tgd or transmasculin* or trans masculin* or tmgnc or gender-nonconform* or gender-non-conform* or gnc or trans people or transpeople or trans person* or transperson or trans m#n or transm#n or trans wom#n or transwom#n or trans feminin* or transfeminin* or transsexual* or female a? birth or male a? birth or afab or amab or female t? male or ftm or male t? female or mtf or non binar* or nonbinar* or genderqueer* or gender queer* or ((gender? or femini#ing or masulini#ing) adj3 hormon*) or gaht or trans sex* or transsex* or gender-expans* or gender-fluid or ((gender or genders or sex or sexual) adj (change or changes or confirmation or expand* or expansive* or reassignment or convers* or alterat* or invers* or affirm* or dysph* or incongru* or divers*))).ti,ab,kf. not ((male t? female or female t? male) adj2 ratio?).ti,ab.	39080
2	exp "sexual and gender minorities"/ or gender-nonconforming persons/ or intersex persons/ or transgender persons/ or Transsexualism/ or exp Gender-Affirming Procedures/ or Gender Dysphoria/ or Health Services for Transgender Persons/	29112
3	1 or 2	52182
4	pain*.ti,ab,kf.	1043954
5	exp Pain/	505571
6	4 or 5	1204112
7	3 and 6	1050
8	(exp animals/ or exp animal experimentation/ or exp models, animal/ or exp plants/ or exp fungi/) not humans/	5968078
9	7 not 8	1032
10	(case reports or editorial or letter or meta analysis or "scoping review" or "systematic review").pt.	4893226
11	9 not 10	864
12	limit 11 to yr="2013 -Current"	776
Link to search		

Embase/Ovid

Search date: June 29, 2026

Embase <1974 to 2026 June 25>		
1	(transident* or trans ident* or transgender* or trans gender* or gender divers* or tgd or transmasculin* or trans masculin* or tmgnc or gender-nonconform* or gender-non-conform* or gnc or trans people or transpeople or trans person* or transperson or trans m#n or transm#n or trans wom#n or transwom#n or trans feminin* or transfeminin* or transsexual* or female a? birth or male a? birth or afab or amab or female t? male or ftm or male t? female or mtf or non binar* or nonbinar* or genderqueer* or gender queer* or ((gender? or femini#ing or masulini#ing) adj3 hormon*) or gaht or trans sex* or transsex* or gender-expans* or gender-fluid or ((gender or genders or sex or sexual) adj (change or changes or confirmation or expand* or expansive* or reassignment or convers* or alterat* or invers* or affirm* or dysph* or incongru* or divers*))).ti,ab,kf. not ((male t? female or female t? male) adj2 ratio?).ti,ab.	51358
2	exp "transgender and gender nonbinary"/ or transsexualism/ or transsexuality/ or exp gender reassignment/ or exp gender affirming hormone therapy/ or exp gender affirming surgery/	28966
3	1 or 2	55477
4	pain*.ti,ab,kf.	1614177
5	exp pain/	2103238
6	4 or 5	2604030
7	3 and 6	2776
8	(exp animal/ or exp invertebrate/ or nonhuman/ or animal experiment/ or animal tissue/ or animal model/ or exp plant/ or exp fungus/) not (exp human/ or human tissue/)	8660783
9	7 not 8	2744
10	((systematic adj3 review?) or meta analy* or metaanaly* or case report*).ti.	1057184
11	9 not 10	2592
12	(preprint or conference abstract or note or letter or editorial).pt.	9755725
13	11 not 12	1527
14	limit 13 to yr="2013 -Current"	1324
		Link to search

Scopus/Elsevier

Search date: June 29, 2026

#1	TITLE-ABS-KEY (pain*)	1966260
#2	TITLE-ABS-KEY (transident* OR "trans ident*" OR transgender* OR "trans gender*" OR "gender divers*" OR tgd OR transmasculin* OR "trans masculin*" OR tmgnc OR "gender-nonconform*" OR "gender-non-conform*" OR gnc OR "trans people" OR transpeople OR "trans person*" OR transperson OR "trans man" OR "trans men" OR transman OR transmen OR "trans woman" OR "trans women" OR transwoman OR transwomen OR "trans feminin*" OR transfeminin* OR transsexual* OR {female at birth} OR {male at birth} OR afab OR amab OR {female to male} OR ftm OR {male to female} OR mtf OR "non binar*" OR nonbinar* OR genderqueer* OR "gender queer*" OR ((gender* OR feminising OR feminizing OR masulinising OR masulinizing) W/3 hormon*) OR gaht OR "trans sex*" OR transsex* OR "gender-expans*" OR "gender-fluid*" OR ((gender OR genders OR sex OR sexual) W/1 (change OR changes OR confirmation OR expand* OR expansive* OR reassignment OR convers* OR alterat* OR invers* OR affirm* OR dysph* OR incongru* OR divers*)))	130196
#3	#1 AND #2	3662
#4	TITLE-ABS ((male PRE/0 to PRE/0 female) W/2 ratio*) OR TITLE-ABS ((female PRE/0 to PRE/0 male) W/2 ratio*)	24357
#5	#3 AND NOT #2	2331
#6	(INDEXTERMS (animals) OR INDEXTERMS ("animal experimentation") OR INDEXTERMS ("models, animal") OR INDEXTERMS (plants) OR INDEXTERMS (fungi)) NOT INDEXTERMS (humans)	7191267
#7	#5 AND NOT #6	2252
#8	TITLE ((systematic W/3 review) OR "meta-analys*" OR metaanalys*) OR DOCTYPE ("ch" OR "cp" OR "ed" OR "no" OR "le" OR "bk" OR "cr" OR "re")	31332408
#9	#7 AND NOT #8	1683
#10	#9 AND PUBYEAR > 2012 AND PUBYEAR < 2027	1401
Search as one string: ((((TITLE-ABS-KEY (pain*)) AND (TITLE-ABS-KEY (transident* OR "trans ident*" OR transgender* OR "trans gender*" OR "gender divers*" OR tgd OR transmasculin* OR "trans masculin*" OR tmgnc OR "gender-nonconform*" OR "gender-non-conform*" OR gnc OR "trans people" OR transpeople OR "trans person*" OR transperson OR "trans man" OR "trans men" OR transman OR transmen OR "trans woman" OR "trans women" OR transwoman OR transwomen OR "trans feminin*" OR transfeminin* OR transsexual* OR {female at birth} OR {male at birth} OR afab OR amab OR {female to male} OR ftm OR {male to female} OR mtf OR "non binar*" OR nonbinar* OR genderqueer* OR "gender queer*" OR ((gender* OR feminising OR feminizing OR masulinising OR masulinizing) W/3 hormon*) OR gaht OR "trans sex*" OR transsex* OR "gender-expans*" OR "gender-fluid*" OR ((gender OR genders OR sex OR sexual) W/1 (change OR changes OR confirmation OR expand* OR expansive* OR reassignment OR convers* OR alterat* OR invers* OR affirm* OR dysph* OR incongru* OR divers*))))) AND NOT (TITLE-ABS ((male PRE/0 to PRE/0 female) W/2 ratio*) OR TITLE-ABS ((female PRE/0 to PRE/0 male) W/2 ratio*))) AND NOT ((INDEXTERMS (animals) OR INDEXTERMS ("animal experimentation") OR INDEXTERMS ("models, animal") OR INDEXTERMS (plants) OR INDEXTERMS (fungi)) NOT INDEXTERMS (humans))) AND NOT (TITLE ((systematic W/3 review) OR "meta-analys*" OR metaanalys*) OR DOCTYPE ("ch" OR "cp" OR "ed" OR "no" OR "le" OR "bk" OR "cr" OR "re")) AND PUBYEAR > 2012 AND PUBYEAR < 2027		

Cochrane Register of Controlled Trials (CENTRAL)/Wiley

Search date: Search date: June 29, 2026

#1	(transident* OR ("trans" NEXT ident*) OR transgender* OR ("trans" NEXT gender*) OR ("gender" NEXT divers*) OR tgd OR transmasculin* OR ("trans" NEXT masculin*) OR tmgnc OR gender-nonconform* OR gender-non-conform* OR gnc OR "trans people" OR transpeople OR ("trans" NEXT person*) OR transperson OR "trans m") NEAR/3 hormon*OR gaht OR ("trans" NEXT sex*) OR transsex* OR gender-expans* OR gender-fluid OR ((gender OR genders OR sex OR sexual) NEXT (change OR changes OR confirmation OR expand* OR expansive* OR reassignment OR convers* OR alterat* OR invers* OR affirm* OR dysph* OR incongru* OR divers*)):ti,ab,kw NOT ((("male" NEXT t? NEXT "female") OR ("female" NEXT t? NEXT "male")) NEAR/2 ratio?):ti,ab	483
#2	[mh "sexual and gender minorities"] OR [mh ^"gender-nonconforming persons"] OR [mh ^"intersex persons"] OR [mh ^"transgender persons"] OR [mh ^Transsexualism] OR [mh "Gender-Affirming Procedures"] OR [mh ^"Gender Dysphoria"] OR [mh ^"Health Services for Transgender Persons"]	760
#3	#1 OR #2	1128
#4	pain*:ti,ab,kw	302287
#5	[mh Pain]	76628
#6	#4 OR #5	310118
#7	#3 AND #6	50
#8	([mh animals] OR [mh "animal experimentation"] OR [mh "models, animal"] OR [mh plants] OR [mh fungi]) NOT [mh ^humans]	1621
#9	#7 NOT #8 with Publication Year from 2013 to 2025, in Trials	47
Link to search		

Sensitivity analysis using untransformed proportions

Sensitivity analyses were performed using untransformed proportions, without application of the Freeman–Tukey double arcsine transformation used in the main analyses.

Sensitivity analysis corresponding to Figure 2A in the main manuscript– Prevalence of pelvic pain among transmasculine individuals receiving testosterone therapy

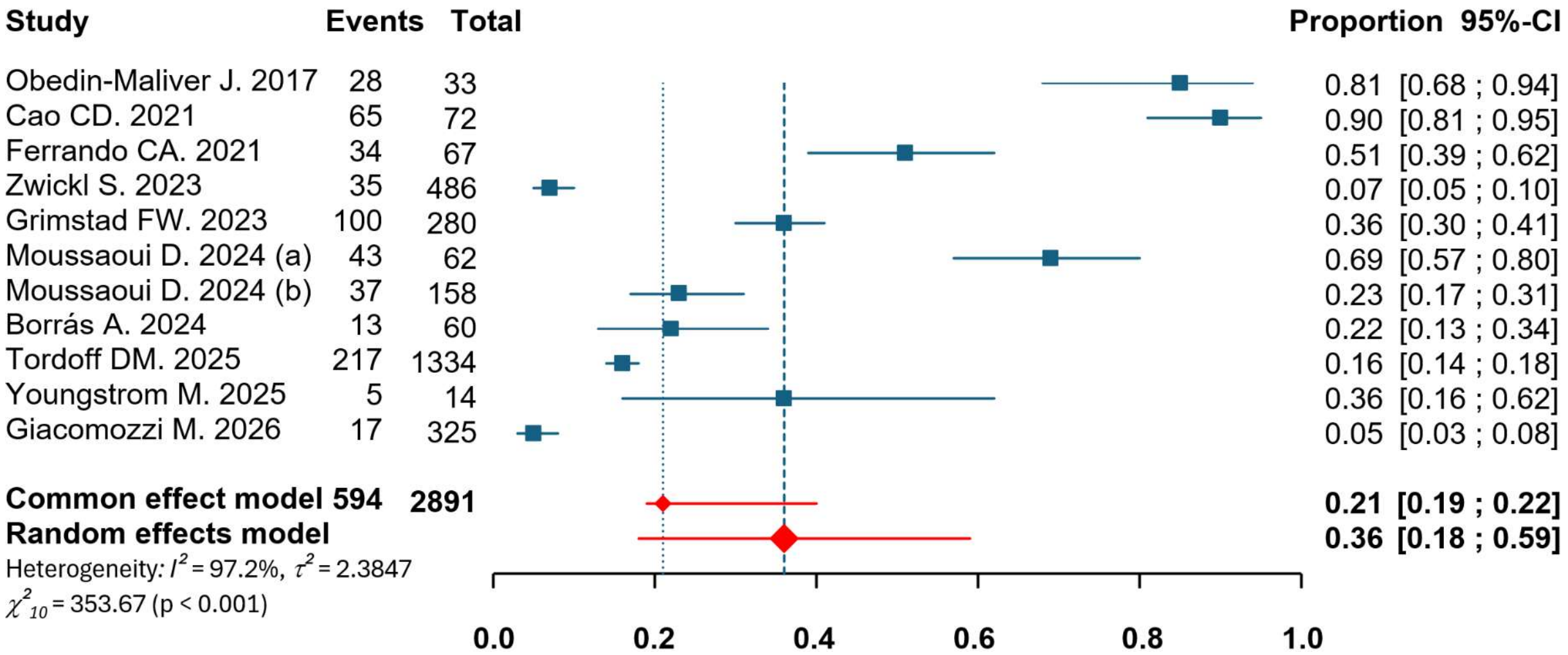

Sensitivity analysis corresponding to Figure 2B in the main manuscript – Prevalence of pelvic pain among transmasculine individuals receiving testosterone therapy, excluding those scheduled for gender-affirming surgery

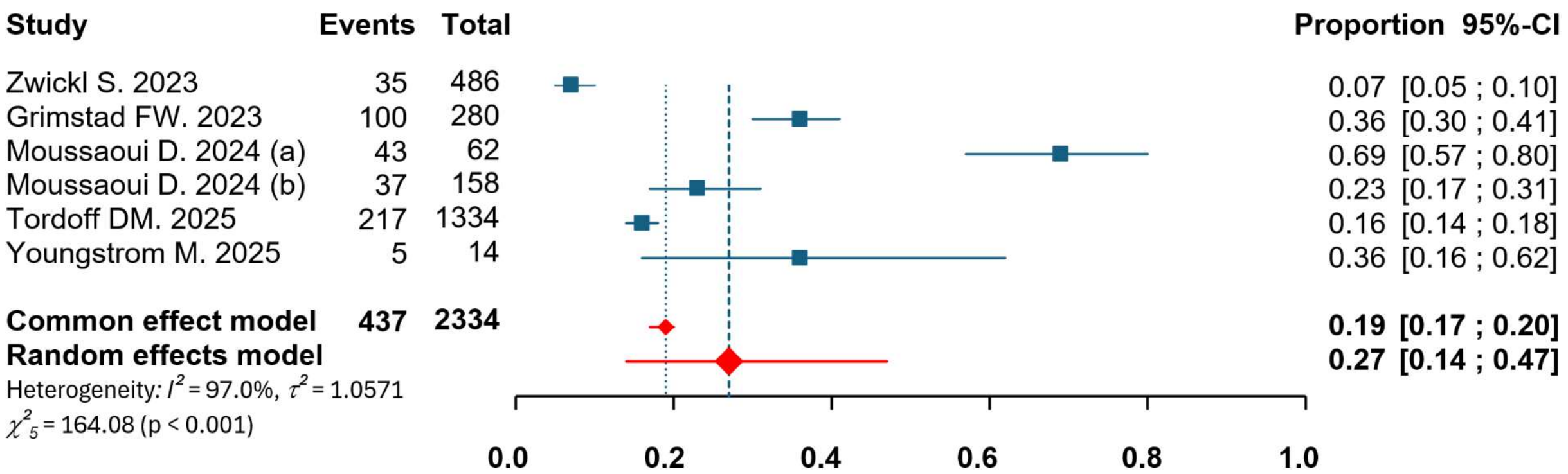

Sensitivity analysis corresponding to Figure 2C in the main manuscript – Prevalence of pelvic pain among transmasculine individuals receiving testosterone therapy and scheduled for gender-affirming surgery

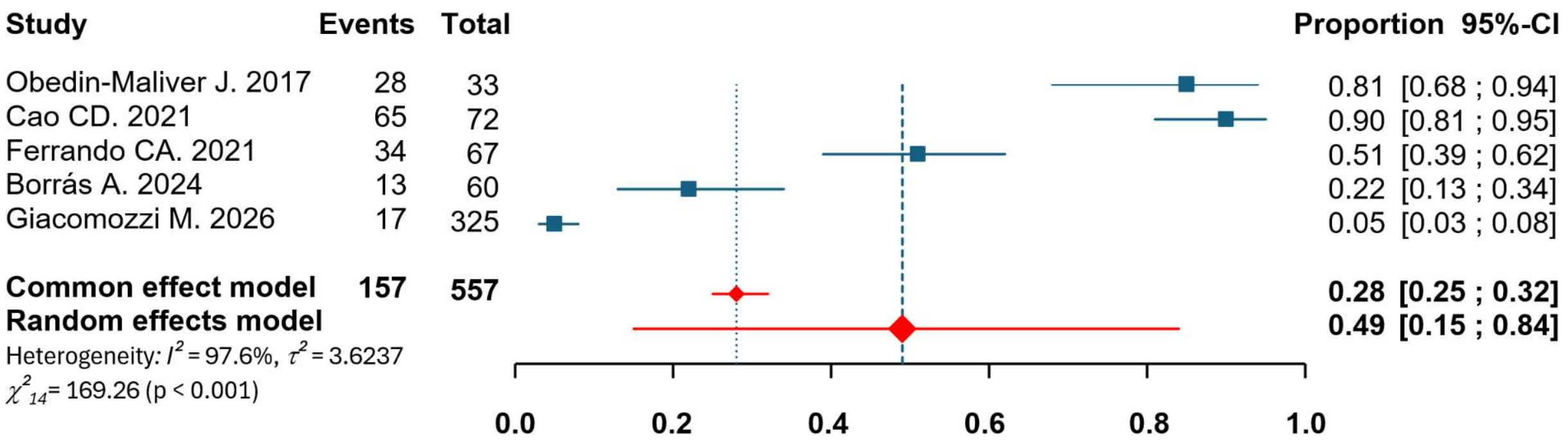

Sensitivity analysis corresponding to Figure 2D in the main manuscript – Prevalence of pelvic pain among transmasculine individuals not receiving testosterone therapy

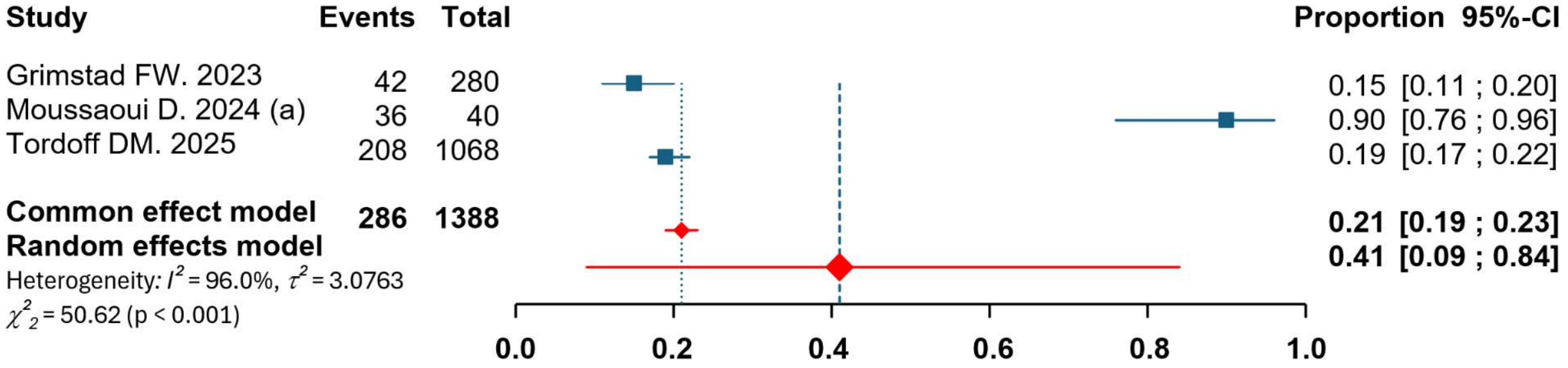

Sensitivity analysis corresponding to Figure 2E in the main manuscript – Proportion of new-onset pelvic pain after testosterone initiation among transmasculine individuals

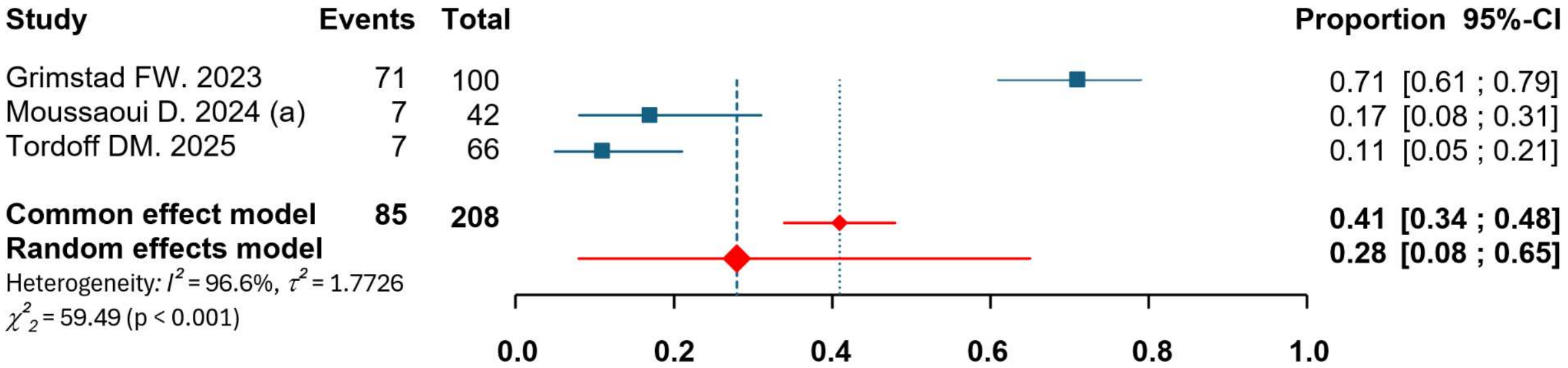

Sensitivity analysis corresponding to Figure 2F in the main manuscript – Prevalence of persistent pelvic pain after testosterone initiation among transmasculine individuals

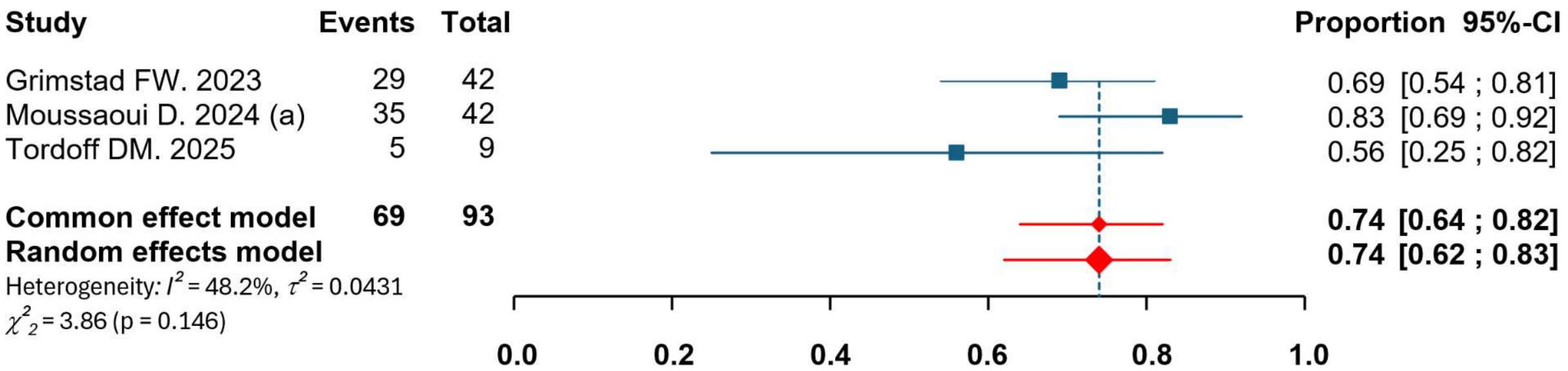

Sensitivity analysis corresponding to Figure 2G in the main manuscript – Prevalence of improved or remitted pelvic pain after testosterone initiation among transmasculine individuals

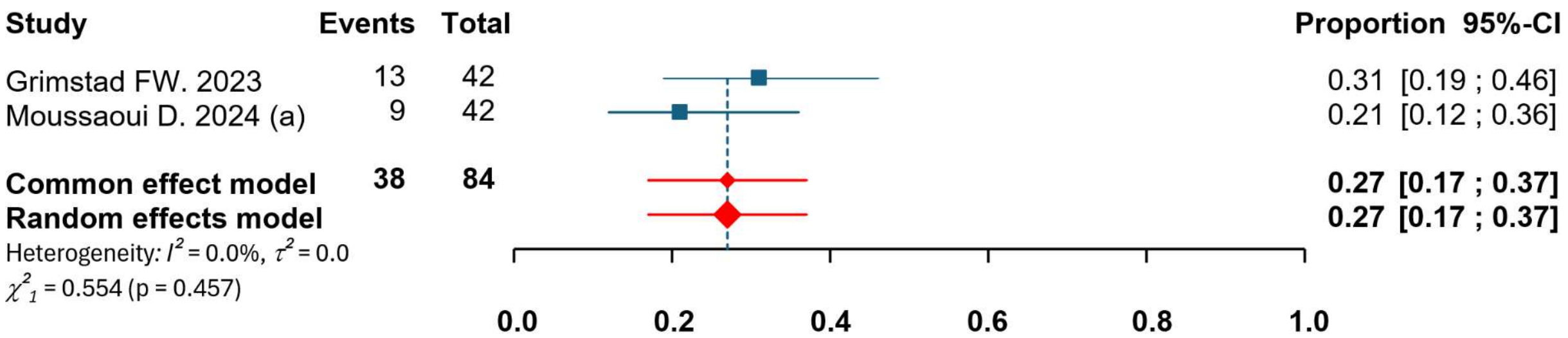

Quality assessment

	Giacomozzi 2026	Tordoff 2025	Moussaoui 2024 (b)	Grimstad 2023	Ferrando 2021	Obedin-Maliver 2017
1. Were the two groups similar and recruited from the same population?	n.a.	n.a.	n.a.	n.a.	n.a.	n.a.
2. Were the exposures measured similarly to assign people to both exposed and unexposed groups?	n.a.	n.a.	n.a.	n.a.	n.a.	n.a.
3. Was the exposure measured in a valid and reliable way?	yes	yes	yes	yes	yes	yes
4. Were confounding factors identified?	yes	yes	yes	yes	yes	yes
5. Were strategies to deal with confounding factors stated?	no	yes	no	no	no	yes
6. Were the groups/participants free of the outcome at the start of the study (or at the moment of exposure)?	n.a.	yes	n.a.	yes	n.a.	n.a.
7. Were the outcomes measured in a valid and reliable way?	yes	yes	yes	yes	yes	yes
8. Was the follow up time reported and sufficient to be long enough for outcomes to occur?	n.a.	yes	yes	n.a	n.a.	yes
9. Was follow up complete, and if not, were the reasons to loss to follow up described and explored?	n.a.	no	n.a.	n.a.	n.a.	n.a.
10. Were strategies to address incomplete follow up utilized?	n.a.	no	n.a.	n.a.	n.a.	n.a.
11. Was appropriate statistical analysis used?	yes	yes	yes	yes	yes	yes
	3/5 **60%**	5/9 **67%**	4/6 **67%**	4/6 **67%**	3/5 **60%**	6/6 **100%**

Joanna Briggs Institute (JBI) Checklist for Cohort Studies

	Zwickl 2023	Moussaoui 2024 (a)	Borrás 2024
1. Were the criteria for inclusion in the sample clearly defined?	yes	yes	yes
2. Were the study subjects and the setting described in detail?	yes	yes	yes
3. Was the exposure measured in a valid and reliable way?	yes	yes	yes
4. Were objective, standard criteria used for measurement of the condition?	yes	yes	yes
5. Were confounding factors identified?	yes	yes	yes
6. Were strategies to deal with confounding factors stated?	no	yes	no
7. Were the outcomes measured in a valid and reliable way?	yes	yes	yes
8. Was appropriate statistical analysis used?	yes	yes	yes
	6/8 **75%**	8/8 **100%**	6/8 **75%**

Joanna Briggs Institute (JBI) Checklist for Analytical Cross-sectional Studies

	Cao 2021
1. Were there clear criteria for inclusion in the case series?	yes
2. Was the condition measured in a standard, reliable way for all participants included in the case series?	yes
3. Were valid methods used for identification of the condition for all participants included in the case series?	yes
4. Did the case series have consecutive inclusion of participants?	yes
5. Did the case series have complete inclusion of participants?	yes
6. Was there clear reporting of the demographics of the participants in the study?	yes
7. Was there clear reporting of clinical information of the participants?	yes
8. Were the outcomes or follow up results of cases clearly reported?	yes
9. Was there clear reporting of the presenting site(s)/clinic(s) demographic information?	yes
10. Was statistical analysis appropriate?	yes
	10/10
	**100%**

Joanna Briggs Institute (JBI) Checklist for Case Series

	Youngstrom 2025
1. Is there congruity between the stated philosophical perspective and the research methodology?	?
2. Is there congruity between the research methodology and the research question or objectives?	yes
3. Is there congruity between the research methodology and the methods used to collect data?	yes
4. Is there congruity between the research methodology and the representation and analysis of data?	yes
5. Is there congruity between the research methodology and the interpretation of results?	yes
6. Is there a statement locating the researcher culturally or theoretically?	?
7. Is the influence of the researcher on the research, and vice- versa, addressed?	no
8. Are participants, and their voices, adequately represented?	yes
9. Is the research ethical according to current criteria or, for recent studies, and is there evidence of ethical approval by an appropriate body?	yes
10. Do the conclusions drawn in the research report flow from the analysis, or interpretation, of the data?	yes
	6/10 **60%**

Joanna Briggs Institute (JBI) Checklist for Qualitative Research
